# The cost-effectiveness of as-needed budesonide-formoterol versus low-dose inhaled corticosteroid maintenance therapy in patients with mild asthma in Canada

**DOI:** 10.1186/s13223-021-00610-w

**Published:** 2021-10-12

**Authors:** Mohsen Sadatsafavi, J. Mark FitzGerald, Paul M. O’Byrne, Mena Soliman, Niroshan Sriskandarajah, Colin Vicente, Sarowar Muhammad Golam

**Affiliations:** 1 Respiratory Evaluation Sciences Program, Collaboration for Outcomes Research and Evaluation, Faculty of Pharmaceutical Sciences, University of British Columbia, 2405 Wesbrook Mall, Vancouver, BC V6T1Z3 Canada; 2 Centre for Lung Health, Vancouver Coastal Health Research Institute, University of British Columbia, 2775 Laurel Street, Vancouver, BC V5Z1M9 Canada; 3 Firestone Institute of Respiratory Health, St Joseph’s Healthcare and Department of Medicine, Michael G. DeGroote School of Medicine, McMaster University, 1280 Main Street West, Health Science Center, 3W10, Hamilton, ON L8S 4K2 Canada; 4 Medical Affairs, AstraZeneca, 1004 Middlegate Road, Mississauga, ON L4Y 1M4 Canada; 5 Market Access, AstraZeneca, 1004 Middlegate Road, Mississauga, ON L4Y 1M4 Canada; 6 PIVINA Consulting Inc., 2600 Skymark Avenue, Suite 11-202, Mississauga, ON L4W 5B2 Canada; 7 Global Market Access and Pricing, BioPharmaceuticals R&D, AstraZeneca, Mölndal, 431 83 Gothenburg, Sweden

**Keywords:** Anti-inflammatory reliever, As-needed, Budesonide-formoterol, Cost-effectiveness, Mild asthma

## Abstract

**Background:**

The Global Initiative for Asthma recommends the use of as-needed low-dose inhaled corticosteroid (ICS)-formoterol as a preferred controller therapy for patients with mild asthma. These recommendations were based, in part, on evidence from the SYGMA 1 and 2 studies of as-needed budesonide-formoterol. This analysis aimed to compare the cost-effectiveness of as-needed budesonide-formoterol to low-dose maintenance ICS plus as-needed short-acting β_2_-agonist (SABA) in patients with mild asthma.

**Methods:**

A Markov cohort model was designed that included three possible health states (non-exacerbation, severe exacerbation, and death) to compare as-needed budesonide-formoterol 200–6 μg to twice-daily budesonide 200 μg maintenance therapy (low-dose ICS) plus as-needed terbutaline 0.5 mg (SABA). The deterministic base-case analysis used severe exacerbation, adverse event (AE), and healthcare resource use data from SYGMA 2, and was conducted from a Canadian public payer perspective with a 50-year time horizon, and a discount rate of 1.5% per annum. Moderate exacerbation was modelled on data from SYGMA 1 in sensitivity analyses. Utility values were derived from SYGMA 2 quality of life data. All-cause- and asthma-related mortality rates and costs (reported in 2019 Canadian dollars) were based on published data, using Canada-specific values where available. One-way deterministic sensitivity, probabilistic sensitivity, and eight scenario analyses were conducted to examine the robustness of the results.

**Results:**

As-needed budesonide-formoterol was the dominant treatment option in the base-case analysis, providing incremental cost savings of $9882 per patient and quality-adjusted life year (QALY) gains of 0.002 versus low-dose maintenance ICS plus as-needed SABA over a 50-year time horizon. Using a willingness-to-pay threshold of $50,000/QALY ($100,000/QALY), as-needed budesonide-formoterol had a 94% (95%) probability of being cost-effective compared with maintenance ICS plus as-needed SABA. Cost-saving was mostly driven by lower overall medication and AE-related costs. As-needed budesonide-formoterol remained the dominant treatment in sensitivity and scenario analyses.

**Conclusions:**

As-needed budesonide-formoterol is a cost-saving option for the treatment of mild asthma from the perspective of the Canadian public payer compared with low-dose maintenance ICS plus as-needed SABA.

**Supplementary Information:**

The online version contains supplementary material available at 10.1186/s13223-021-00610-w.

## Background

Asthma is a chronic inflammatory disease of the airways, which is of significant public health concern in Canada [1]; approximately 2.6 million Canadians aged ≥ 12 years were living with asthma in 2018, representing 8.3% of the population [1, 2]. There is a substantial economic burden associated with asthma, and it has been estimated that sub-optimal asthma control will cost $213 billion and result in a loss of 1.38 million quality-adjusted life years (QALY) in Canada between 2014 and 2033 [3]. Mild asthma is common, occurring in 50–75% of patients with asthma [4], and although patients with more severe disease are responsible for the largest proportion of healthcare resource use [5], a considerable proportion of healthcare resource use is also attributable to patients with poorly controlled mild asthma [5, 6].

Previous Global Initiative for Asthma (GINA) reports have recommended daily maintenance inhaled corticosteroids (ICS) with short-acting β_2_-agonists (SABA) reliever therapy for the treatment of mild asthma [7]. However, real-world adherence to maintenance ICS treatment is typically poor, with patients tending to rely on SABA to manage their symptoms [8–10]. Overuse of SABA is prevalent [8, 9, 11], and monotherapy with SABA, i.e. without concomitant ICS use, is associated with an increased risk of exacerbations [9, 11, 12]. In Canada, inappropriate and excessive use of SABA has been observed [13, 14], with one study estimating that 28% of patients used at least three SABA canister refills per year without concomitant ICS prescriptions [14], and another suggesting up to a 1.4-fold increase in risk of annual exacerbations when SABA use is ≥ 3 canister refills per year (versus ≤ 2 refills per year) [15].

Given these risks, and the lack of an anti-inflammatory effect with SABA alone as initial therapy in mild asthma [10], the Canadian Thoracic Society guidelines define overuse of SABA as > 2 canisters in a year [16]. Daily ICS plus as-needed SABA is recommended as first line controller therapy in mild asthma, with as-needed budesonide-formoterol recommended as an alternative in patients ≥ 12 years with poor adherence to daily medication despite substantial asthma education and support [16]. Due to the risk of low adherence to the ICS component (thus subjecting the patient to SABA monotherapy) in previous guidelines, the latest GINA report now recommends symptom-driven as-needed low-dose ICS in combination with the long-acting β_2_-agonist (LABA), formoterol, as the preferred reliever for patients ≥ 12 years requiring GINA Step 1–2 treatment, i.e., when symptoms occur up to 4–5 days per week [17].

This therapeutic approach is supported by the results of two pivotal 52-week, double-blind, randomized controlled trials, SYGMA 1 [18] and SYGMA 2 [19], in patients with mild asthma, and two open-label, 52-week studies in patients with mild-to-moderate asthma (PRACTICAL) [20] or mild asthma (Novel START) [21], as well as a complementary systematic review and meta-analysis [22]. SYGMA 1, SYGMA 2 and Novel START demonstrated a comparable annual rate of severe exacerbations between as-needed budesonide-formoterol and twice daily budesonide maintenance (low-dose ICS) plus as-needed terbutaline (SABA), whereas the rate was significantly lower with as-needed budesonide-formoterol in the PRACTICAL study [20]. In the two studies that included an as-needed SABA arm, SYGMA 1 [18] and Novel START [21], the exacerbation rate was significantly lower with as-needed budesonide-formoterol than as-needed terbutaline.

On the basis of this evidence, budesonide-formoterol as anti-inflammatory reliever therapy was approved in Canada in September 2019 for patients aged ≥ 12 years with mild persistent asthma [23, 24]. A recent study found that as-needed budesonide-formoterol is cost-effective versus maintenance ICS plus as-needed SABA from a UK healthcare payer’s perspective [25], but this strategy has not been assessed from a Canadian perspective.

Canada has a public healthcare system that is managed by provincial and territorial governments to deliver medically necessary healthcare services to all legal residents. However, medication costs are covered only for those on low income, those enrolled in income-based universal programs, or other population groups that may require enhanced coverage for higher drug costs [26]. For the rest of the population, medication costs are paid either out-of-pocket or through private (usually employer) health insurance. For patients with chronic respiratory conditions this can represent an onerous economic burden.

In Canada, in addition to clinical efficacy data, information on the economic value of health technologies (e.g., medications) is a formal requirement for the adoption of such technologies into the healthcare system. Therefore, the primary objective of this study was to compare the cost-effectiveness of as-needed budesonide-formoterol to low-dose maintenance ICS plus as-needed SABA from a Canadian public payer perspective in patients with mild asthma.

## Materials and methods

This study is reported according to the Consolidated Health Economic Evaluation Reporting Standards guidelines [27].

We developed a discrete-time Markov model to simulate the trajectory of mild asthma and the impact of treatment. A Markov cohort model is a well-accepted method for modelling asthma outcomes and their associated costs and impact on quality of life [28–30].

### Patient population

The eligible patient population was that of the SYGMA studies [18, 19], i.e., asthma patients ≥ 12 years of age with a documented clinical diagnosis of mild asthma (GINA 2012 criteria [31]), either uncontrolled on as-needed SABA alone or controlled on regular low-dose ICS or a leukotriene receptor antagonist plus as-needed SABA.

### Cost-effectiveness model

**Fig. 1:**
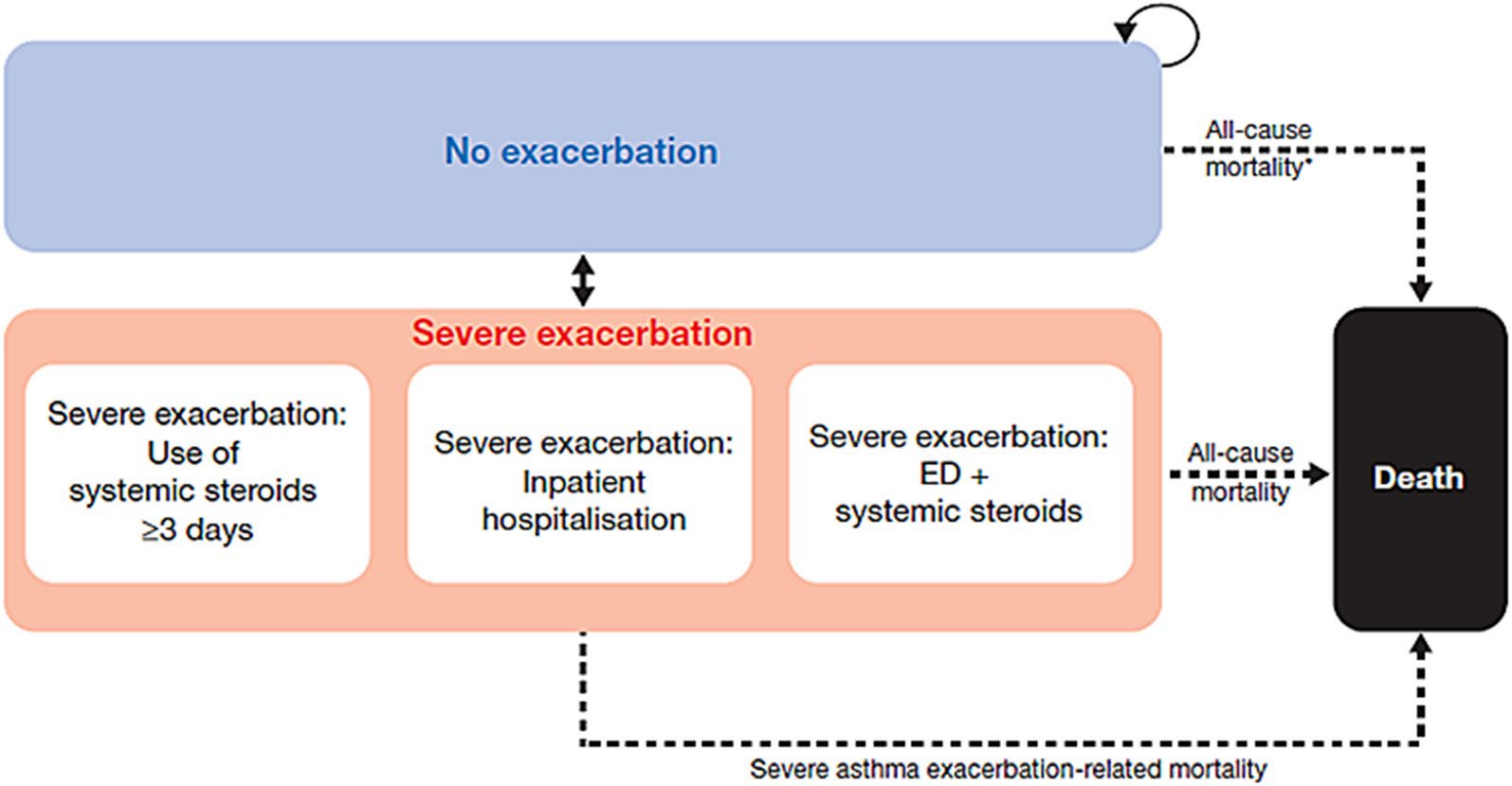
Base-case model structure. ED: emergency department

The Markov model had three health states (Fig. 1): (i) non-exacerbation; (ii) severe exacerbation; and (iii) death. Consistent with the American Thoracic Society/European Respiratory Society definition, a severe exacerbation was defined as a deterioration of asthma requiring the use of systemic corticosteroids (SCS) for at least 3 days, an emergency department (ED) visit resulting in SCS treatment, or inpatient care [32]. Moderate exacerbations data, sourced from SYGMA 1, were only included in the sensitivity analyses; such events were defined as a deterioration of asthma requiring a change in prescribed treatment, i.e., initiation of additional ICS to avoid progression to a severe exacerbation [18].

This model was designed from a public payer perspective, with only direct healthcare costs considered in the base-case analysis. We adopted a time horizon of 50 years to cover the lifetime of the majority of patients. The model used weekly cycles, reflecting the duration of asthma exacerbations observed in SYGMA 2 [19]. A discount rate of 1.5% per annum for both costs and outcomes was applied, as per the Canadian Agency for Drugs and Technologies in Health (CADTH) guidelines [33].

All patients entered the model in the non-exacerbation health state. Healthcare resource utilization data and health-related quality of life data from SYGMA 2 provided inputs for the model in this state. During each cycle of the model, patients either transitioned to a new health state or remained in their current state, with transitions according to a probability distribution derived from clinical data from SYGMA 2. Patients remained in the exacerbation health state for one cycle before transitioning to one of the other health states. Transitions to death had two components of risk: asthma-related risk of death and population level age- and gender-specific risk of death (background mortality) in Canada. In the sensitivity analyses, when the withdrawal health state was used, patients who transitioned to the withdrawal health state remained there for the rest of the time horizon or until death.

Results are presented as incremental costs per patient, incremental QALY per patient, and the incremental costs per QALY gained. All costs were reported in 2019 Canadian dollars ($). The inputs to the base-case model, as well as the main variables tested in the sensitivity analyses, are summarized in Table 1.

**Table 1:** Summary of Markov Model parameters included in the base-case and one-way deterministic sensitivity analyses

	Base-case	Value in DSA, lower/upper	PSA distribution used (justification)	Source
Baseline characteristics				
Age at start, mean	41.0 years	32.8/49.2 years	Gamma (Likely skewed nature of age, and its constraint to positive values)	SYGMA 2 [19]
Annual severe exacerbation rate				
As-needed budesonide-formoterol (95% CI)	0.11 (0.10–0.13)	0.088/0.132	Beta (α = 88.89, β = 719.20)	SYGMA 2 [19]
Low-dose maintenance ICS + as-needed SABA (95% CI)	0.12 (0.10–0.14)	0.096/0.144	Beta (α = 87.88, β = 644.45)	
Distribution of patients by severe exacerbation health states				
As-needed budesonide-formoterol				
SCS	81.9%		Dirichlet (The percentages across severe exacerbation management states must sum to 100%)	
ED + SCS	10.2%			SYGMA 2 [19]
Inpatient hospitalization	7.9%			
Low-dose maintenance ICS + as-needed SABA				
SCS	78.4%			
ED + SCS	15.2%			
Inpatient hospitalization	6.4%			
Annual moderate exacerbation rate				
As-needed budesonide-formoterol	Not included	0.07	N/A	SYGMA 1 [18]
Low-dose maintenance ICS + as-needed SABA	Not included	0.06		
Annual rate of withdrawal				
As-needed budesonide-formoterol	Not included	SYGMA 1: 0.8%, SYGMA 2: 0.7%	Dirichlet (The percentages across severe exacerbation management states must sum to 100%)	SYGMA 1 [18], SYGMA 2 [19]
Low-dose maintenance ICS + as-needed SABA	Not included	SYGMA 1: 1.2%, SYGMA 2: 1.1%		
Annual proportion of adverse events				
As-needed budesonide-formoterol vs low-dose maintenance ICS + as-needed SABA		N/A		SYGMA 2 [19]
Viral URTI	7.4% vs. 8.0%		Beta (α = 95.53, β = 1157.83) vs. (α = 91.92, β = 1057.08)	
URTI	3.9% vs. 4.3%		Beta (α = 96.06, β = 2367.04) vs. (α = 95.66, β = 2128.92)	
Bronchitis	3.1% vs. 3.7%		Beta (α = 96.87, β = 3027.94) vs. (α = 96.26, β = 2505.44)	
Pharyngitis	2.4% vs. 3.0%		Beta (α = 97.58, β = 3968.09) vs. (α = 96.97, β = 3135.36)	
Headache	2.5% vs. 2.4%		Beta (α = 97.48, β = 3801.53) vs. (α = 97.58, β = 3968.09)	
Allergic rhinitis	2.4% vs. 2.1%		Beta (α = 97.58, β = 3968.09) vs. (α = 97.88, β = 4563.03)	
Influenza	1.6% vs. 2.1%		Beta (α = 98.38, β = 6050.62) vs. (α = 97.88, β = 4563.03)	
Medication, inhalations/day				
As-needed budesonide-formoterol	0.52	N/A	Gamma (α = 100, β = 0.0052)	SYGMA 2 [19]
Low-dose ICS	2.00	1.60/2.40	Gamma (α = 100, β = 0.02)	Fluticasone PI [54]
As-needed SABA when combined with low-dose ICS	0.49	N/A	Gamma (α = 100, β = 0.0049)	SYGMA 2 [19]
Direct medical costs				
Severe exacerbation, per event^a^			Gamma (Likely skewed nature of healthcare costs, and their constraint to positive values)	SYGMA 2 [19]
SCS	$155.14	$124.11/$186.16		
ED + SCS	$490.81	$392.64/$588.97		
Inpatient hospitalization	$9399.94	$7519.95/$11,279.92		
Non-exacerbation health state (weekly)	$38.40	$30.72/$46.08		
Adverse events				
Viral URTI	$7909.00			
URTI	$7909.00			
Bronchitis	$157.00			
Pharyngitis	$157.00			
Headache	$157.00			
Allergic rhinitis	$157.00			
Influenza	$6038.00			
Drug acquisition costs, per day				
As-needed budesonide-formoterol	$0.39	N/A	Fixed^b^	SYGMA 2 [19]
Low-dose maintenance ICS	$0.72	N/A		
As-needed SABA for patients on low-dose maintenance ICS	$0.01	N/A		
Utility inputs for health states^c^				
Utility of non-exacerbation	0.867	0.694/1.000	Beta (α = 12.43, β = 1.91)	SYGMA 2 (EQ-5D) [19]
Disutility of severe exacerbation				
SCS	− 0.10	− 0.08/− 0.12	− Beta^d^ (α = 110.1, β = 1211.1)	Lloyd 2006[44]
ED + SCS	− 0.15	− 0.12/− 0.18	− Beta (α = 115.15, β = 882.8)	Assumption
Inpatient hospitalization	− 0.20	− 0.16/− 0.24	− Beta (α = 120.2, β = 721.2)	Lloyd 2006[44]
Disutility of moderate exacerbation	Not included	− 0.04/− 0.06	− Beta (α = 105.05, β = 2206.05)	Assumption^e^
Discount rate^f^	1.5%	0.0%/3.0%	N/A	

CI, confidence interval; DSA, deterministic sensitivity analysis; ED, emergency department; EQ-5D, EuroQoL-5 dimension, ICS, inhaled corticosteroid; PSA, probabilistic sensitivity analysis; PI, prescribing information; N/A, not applicable; SABA, short-acting β_2_-agonist; SCS, systemic corticosteroid; URTI, upper respiratory tract infection

^a^Derivation of severe exacerbation event costs is described in Additional file 1: Table S2. The weekly cost of the non-exacerbation health state was added to the severe exacerbation health state costs

^b^Drug acquisition costs were assumed to be known and fixed

^c^Disutility values for adverse events were assumed to only affect patients within the non-exacerbation health state to avoid double counting

^d^Disutility values were sampled from beta and then the negative value was taken

^e^The utility value for moderate exacerbations was assumed to be half of a severe exacerbation requiring SCS only (i.e., − 0.05)

^f^For both costs and outcomes

### Treatment comparisons

The Canadian Thoracic Society guidelines recommend the use of SABA as a reliever in individuals with mild asthma on ICS monotherapy [16], which is the same as the comparator group in the SYGMA studies. The treatment groups in SYGMA 1 and SYGMA 2 from which clinical data were sourced for this analysis were the as-needed budesonide-formoterol 200–6 μg (Symbicort^®^ Turbuhaler^®^, AstraZeneca) group, with 1277 patients in SYGMA 1 and 2089 in SYGMA 2, and the twice-daily budesonide 200 μg maintenance therapy (low-dose ICS; Pulmicort^®^ Turbuhaler^®^, AstraZeneca) plus as-needed terbutaline 0.5 mg (SABA; Turbuhaler^®^, AstraZeneca) group, with 1282 patients in SYGMA 1 and 2087 in SYGMA 2 [18, 19].

### Source of clinical data

Clinical efficacy evidence for the base-case analysis was derived from SYGMA 2 [19]. Inputs were annual severe exacerbation rates, stratified by three different severe exacerbation types (use of SCS; ED visit plus SCS; inpatient hospitalization—based on resources used), and proportions of adverse events (AEs) (Table 1). The relative frequency of severe exacerbations in SYGMA 2 was as follows: (i) use of SCS: 81.9% with as-needed budesonide-formoterol versus 78.4% with budesonide maintenance plus as-needed terbutaline; (ii) ED visit plus SCS: 10.2% versus 15.2%, respectively; and (iii) inpatient hospitalization: 7.9% versus 6.4%, respectively.

The most common AEs in SYGMA 2 (occurring in ≥ 2% of patients) were used: viral upper respiratory tract infection (viral URTI), URTI, bronchitis, pharyngitis, headache, allergic rhinitis, and influenza. Annual risk of AEs for each treatment were considered relevant to the model for patients in the non-exacerbation health state. To test the robustness of the results to variations in AE rates, a scenario analysis was completed that assigned no AEs to each comparator.

### Treatment effect

The annual rates of severe exacerbation in SYGMA 2 were 0.11 [95% confidence interval (CI): 0.10–0.13] with as-needed budesonide-formoterol and 0.12 (95% CI 0.10–0.14) with budesonide maintenance plus as-needed terbutaline [19]. To convert the annual exacerbation rates from SYGMA 2 to transition probabilities, they were recalculated as a weekly rate and then converted to weekly probabilities using the formula *p* = 1-exp(-*r*), where *p* is probability and *r* is the rate [34]. In extrapolating treatment effects beyond the follow-up time of the SYGMA studies, we assumed a fixed treatment effect, with no effect waning over time. The distribution of the model cohort among the three severe exacerbation types remained the same over the time horizon of the model.

### Source of mortality data

This analysis accounted for asthma-related and all-cause mortality. General population mortality data adjusted by age and gender were sourced from Canadian life tables [35]. It was assumed that asthma-related mortality can only occur from the exacerbation state. Deaths due to asthma exacerbations were modelled based on previously published data [36–38]. For exacerbations requiring hospital admission, this cost-effectiveness analysis used mortality data from Watson et al*.* [37] combined with Roberts et al. [36], and for exacerbations not requiring a hospital admission (i.e., ED visit and use of SCS) from Watson et al*.* [37], combined with data from the National Review of Asthma Deaths (NRAD) 2017 [38]. The annual risk of death related to severe asthma exacerbations is summarized in Additional file 1: Table S1. Annual risks were converted to a weekly risk and added to the risk of death for the general population.

### Resource use and costs

We used the Ontario Drug Benefit Formulary/Comparative Drug Index (May 2020) to derive drug acquisition costs [39]. Derivation of total drug acquisition costs per day in each treatment group is shown in Table 2; costs per day for ICS maintenance therapy were based on the prescribed dose (i.e., two inhalations per day) of the most commonly prescribed maintenance ICS and SABA reliever for mild asthma in Canada (fluticasone propionate and salbutamol, respectively [40]). Based on best practice recommendations [33, 41], ICS maintenance therapy costs were calculated assuming full utilization (Table 2), therefore a scenario analysis was conducted to test whether costs based on actual maintenance ICS utilization as reported in SYGMA 2 (i.e. mean percentage of daily doses 62.8% [19]) influenced the outcome of the analysis.

**Table 2:** Drug acquisition costs (2019), Canadian $ [39]

Therapy	Inhaler	Cost per inhaler ($)	Inhalations per inhaler	Inhalations per day	Cost per day ($)
As-needed budesonide-formoterol	Symbicort^®^ Turbuhaler^®^	$90.36	120	0.52 (SYGMA 2)	0.39
Low-dose maintenance ICS + as-needed SABA	Salbutamol 0.4 mg (Ventolin^®^)	$5.00	200	0.49^a^	0.01
	Fluticasone 125 μg (Flovent^®^)	$43.00	120	2^b^	0.72

ICS, inhaled corticosteroid; SABA, short-acting β_2_-agonist

^a^Mean number of inhalation per day based on SYGMA 2 [19]

^b^Prescribed (as labelled) number of inhalations per day [54]

The costs of severe exacerbations were calculated for each exacerbation type using healthcare resource utilization data from SYGMA 2 and Canadian unit costs (Additional file 1: Table S2). Specifically, SCS cost per day was calculated using the unit cost of prednisolone for a mean daily dose of 45 mg multiplied by the mean number of days SCS was used in SYGMA 2. The cost of an ED visit plus SCS was calculated using the unit cost of an ED visit ($412.87) added to SCS costs per day, calculated as described above. Hospitalization costs per stay ($9322) were calculated using weighted 2019 costs from the Ontario Case Costing Initiative Cost Analysis Tool (Code J441) [42], and mean length of stay in SYGMA 2 (6.9 days). The weekly costs of the non-exacerbation health state were added to the severe exacerbation costs, as it was assumed that patients experiencing a severe asthma exacerbation would still incur the weekly monitoring costs related to non-exacerbation. Healthcare costs associated with moderate exacerbations also included weekly monitoring costs as well as direct exacerbation costs.

Non-exacerbation health state costs were based on healthcare resource utilization data from SYGMA 2 (e.g., number of specialist, ED and general practitioner visits), and associated standard specific unit costs in 2017 [43] (Additional file 1: Table S3). Costs associated with the non-exacerbation health state were included in the base-case analysis (Table 1).

### Sources of utility data

Utility data were collected in SYGMA 2 using the EuroQoL-5 Dimension-5 Level, and were used to provide utility weights in the model. The utility value for the non-exacerbation health state in the base-case analysis was 0.867, taken from the SYGMA 2 trial for patients not experiencing any exacerbation (Table 1). Severe exacerbations were associated with a decrement of utility (disutility), which was derived from a previous study by Lloyd et al*.* [44]. The study did not include a disutility value for a severe exacerbation requiring an ED visit plus SCS, so this was assumed to be − 0.15, the midway between the utility among patients requiring the use of SCS (− 0.10) and those requiring inpatient hospitalization (− 0.20) (Table 1). The disutility of the exacerbation was applied for the duration of the exacerbation.

### Treatment withdrawal

Withdrawal was defined as discontinuation of treatment due to development of study-specific withdrawal criteria or due to AEs. Withdrawal was not included in the base-case analysis but it was included in the sensitivity analysis where withdrawal health states included step up from SABA alone to GINA Step 2 (daily low-dose ICS plus as-needed SABA or as-needed low-dose ICS-formoterol), and from Step 2 to Step 3 (low-dose ICS-LABA plus as-needed SABA or as-needed low-dose ICS-formoterol for patients prescribed maintenance and reliever therapy) [45]. Annual risks of withdrawal used in the sensitivity analysis were derived from SYGMA 1 and SYGMA 2 (0.8% and 0.7% with as-needed budesonide-formoterol, respectively, versus 1.2% and 1.1% with maintenance ICS plus as-needed SABA) (Table 1).

### Analyses

#### Deterministic base-case analysis

The base-case analysis was based on point estimates of the previously described parameters. The main outcomes were total discounted costs and QALY with as-needed budesonide-formoterol compared with low-dose ICS plus as-needed SABA, as well as incremental costs and QALY. From these, the incremental costs per QALY was calculated to determine the dominant treatment strategy.

#### Probabilistic sensitivity analysis

Probabilistic sensitivity analysis (PSA) was performed using Monte Carlo simulations to incorporate uncertainty in the evidence base, involving the use of 1000 iterations. The methodology for assigning probability distributions for these simulations is described in Table 1. Where the information characterizing the parameter distribution was not available, we assumed a variance-to-mean ratio of 10%. PSA outcomes are shown in the form of a cost-effectiveness plane and cost-effectiveness acceptability curves (CEACs). The former depicts the joint distribution of incremental costs and QALY, whereas the latter presents the probability of cost-effectiveness at a range of willingness-to-pay (WTP) values for one QALY.

#### Deterministic sensitivity analyses

One-way deterministic sensitivity analyses (DSA) were performed to identify variables that influenced model results. In one analysis, the discount rate was changed to 0% and 3% (base-case 1.5%). Additionally, different values were examined for cohort starting age, severe exacerbation rates, cost units, ICS dose (inhalations/day), and (dis)utility values. Annual rates of withdrawal were also included in the one-way DSA. Finally, in a separate analysis, we included moderate exacerbations and associated cost and disutility values, based on data from SYGMA 1 (Table 1).

#### Scenario analyses

A series of probabilistic scenario analyses were performed to determine the robustness of the results to variations in several parameters at once. These analyses, which utilized 1000 PSA iterations, were as follows: Scenario 1 = discount rate of 0% for costs and benefits; Scenario 2 = discount rate of 3.0% for costs and benefits; Scenario 3 = time horizon of 2 years; Scenario 4 = time horizon of 10 years; Scenario 5 = societal perspective; and Scenario 6 = budesonide (Pulmicort®) as the ICS in the low-dose maintenance ICS plus as-needed SABA group; Scenario 7 = set utilization rate of ICS to 62.8%, as per the ICS maintenance group in SYGMA 2; and Scenario 8 = removal of AEs from the analysis.

## Results

### Base-case analysis

The estimated discounted total costs of treatment per patient over a 50-year time horizon were $36,439 with as-needed budesonide-formoterol and $46,321 with low-dose maintenance ICS plus as-needed SABA. The largest difference in costs were drug acquisition costs per day and AE-related costs (Table 3). Discounted QALY over a 50-year time horizon were 25.923 with as-needed budesonide-formoterol and 25.921 with maintenance ICS treatment plus as-needed SABA. Therefore, as-needed budesonide-formoterol was the dominant treatment, given its incremental cost savings of $9882 per patient and QALY gains of 0.002 versus maintenance ICS plus as-needed SABA over the 50-year time horizon.

**Table 3:** Summary of comparison of costs and QALYs (deterministic base-case), Canadian $

Parameter	Cost: as-needed budesonide-formoterol	Cost: low-dose maintenance ICS + as-needed SABA	Incremental Cost ($)	QALY: as-needed budesonide-formoterol	QALY: low-dose maintenance ICS + as-needed SABA	Incremental QALYs
Non-exacerbation	$1146.16	$1145.89	$0.27	25.875	25.869	0.006
Severe exacerbation	$3010.65	$2847.82	$162.83	0.047	0.052	− 0.004
Drug costs	$4268.32	$7943.94	− $3675.62	N/A	N/A	N/A
Adverse events	$28,013.94	$34,384.33	− $6370.39	0.000	0.000	0.000
Total	$36,439.08	$46,321.98	− $9882.90	25.923	25.921	0.002

Withdrawal, moderate exacerbations and death-related costs were not included in this base-case analysis

ICS, inhaled corticosteroid; N/A, not applicable; QALY, quality-adjusted life year; SABA, short-acting β_2_-agonist

### Probabilistic sensitivity analysis

**Fig. 2:**
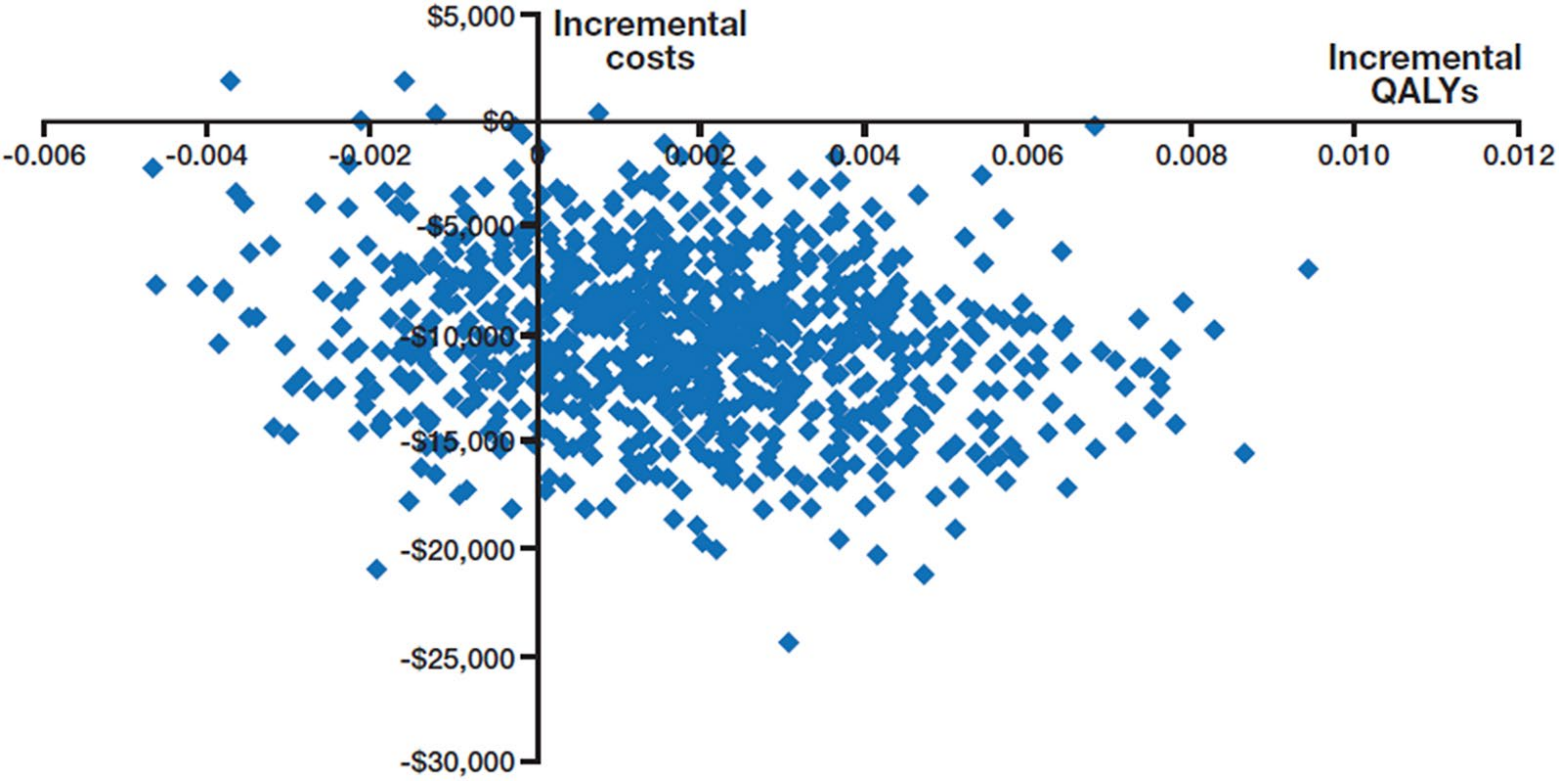
Cost-effectiveness plane for as-needed budesonide-formoterol versus low-dose ICS plus as-needed SABA. ICS: inhaled corticosteroid; QALY: quality-adjusted life year; SABA: short-acting β_2_-agonist

**Fig. 3:**
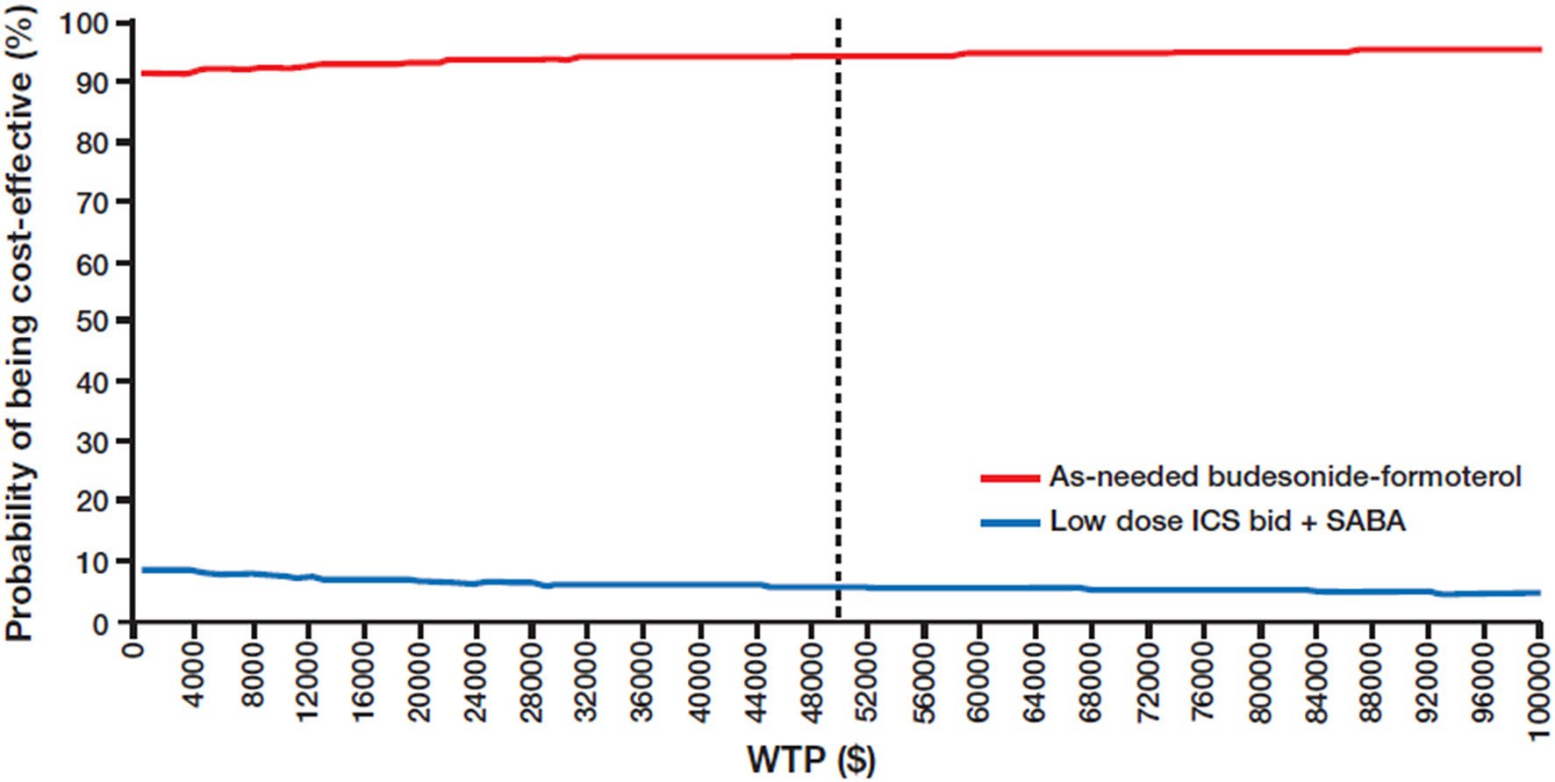
Cost-effectiveness acceptability curve for as-needed budesonide-formoterol versus low-dose maintenance ICS plus as-needed SABA. Vertical line on graph indicates WTP threshold of $50,000. bid: twice daily; ICS: inhaled corticosteroid; SABA: short-acting β_2_-agonist; WTP: willingness-to-pay threshold

The results of the PSA are shown in Fig. 2. The CEACs (Fig. 3) showed that the probability of as-needed budesonide-formoterol being cost-effective, i.e., of being within the WTP threshold of $50,000 per QALY gained compared with maintenance ICS plus as-needed SABA was 94%. The probability of being within a WTP threshold of $100,000 per QALY was 95%.

### Deterministic sensitivity analysis

As-needed budesonide-formoterol was the dominant treatment strategy in all variations assessed in the one-way DSA; although the impact on the model differed for each variable studied, the model was not sensitive to any one parameter.

### Scenario analyses

As-needed budesonide-formoterol was the dominant treatment strategy in all eight of the scenarios shown in Table 4, including shorter time horizons [2 or 10 years] and lower (0%) or higher (3.0%) discount rates than in the base-case analysis. As-needed budesonide-formoterol was dominant over the shorter time horizons because, although QALY gains were equal between treatment groups (both 0.000), the incremental cost savings per patient were $662 over a time horizon of 2 years and $3061 over 10 years. Incremental cost savings and QALY gains from the societal perspective with as-needed budesonide-formoterol were broadly similar to that observed in the base-case analysis, with an incremental cost saving of $9882 per patient and QALY gains of 0.002 versus maintenance ICS plus as-needed SABA. With budesonide as the ICS in the low-dose maintenance ICS group, as-needed budesonide-formoterol was associated with a cost saving of $14,431 and a QALY gain of 0.002. Moreover, as-needed budesonide-formoterol continued to be cost-saving and produce additional QALY gains in the scenarios that adjusted the utilization rate of ICS and removed AE rates.

**Table 4:** Scenario analyses

	Total cost		Total QALY		ICER		
	As-needed budesonide-formoterol	Low-dose maintenance ICS + as-needed SABA	As-needed budesonide-formoterol	Low-dose maintenance ICS + as-needed SABA	Incremental cost	Incremental QALY	Dominant strategy
Scenario 1: discount rate 0%	$48,996	$62,385	34.579	34.576	− $13,388	0.003	As-needed budesonide-formoterol
Scenario 2: discount rate 3%	$28,346	$35,890	20.136	20.135	− $7544	0.001	As-needed budesonide-formoterol
Scenario 3: time horizon 2y	$2417	$3078	1.723	1.723	− $662	0.000	As-needed budesonide-formoterol
Scenario 4: time horizon 10y	$11,338	$14,398	8.093	8.093	− $3061	0.000	As-needed budesonide-formoterol
Scenario 5: societal perspective	$37,387	$47,269	25.960	25.958	− $9882	0.002	As-needed budesonide-formoterol
Scenario 6: budesonide as low-dose maintenance ICS	$36,546	$50,976	26.030	26.028	− $14,431	0.002	As-needed budesonide-formoterol
Scenario 7: ICS utilization adjusted to 62.8% (fixed)	$36,700	$43,606	25.177	25.175	− $6906	0.002	As-needed budesonide-formoterol
Scenario 8: removal of AEs from the analysis	$8449	$12,002	25.369	25.367	− $3552	0.002	As-needed budesonide-formoterol

AE, adverse event; ICER, incremental cost-effectiveness ratio; ICS, inhaled corticosteroid; QALY, quality-adjusted life year; SABA, short-acting β_2_-agonist; y, year

## Discussion

In this economic evaluation, as-needed budesonide-formoterol was the dominant treatment option compared with low-dose maintenance ICS therapy plus as-needed SABA in patients with mild asthma over a 50-year time horizon from a Canadian public payer perspective, with costs savings of $9882 per patient. These findings support those of a cost-effectiveness analysis from the UK healthcare payer perspective, which made similar treatment comparisons based on SYGMA 2 data [25].

An important finding in both the SYGMA 1 and SYGMA 2 studies is observed treatment adherence. In the budesonide maintenance groups, 79% of patients in SYGMA 1 were adherent to daily ICS [18]; this value was 63% in SYGMA 2, although daily adherence reminders were not given, unlike in SYGMA 1 [19]. These values are much higher than real-world ICS adherence rates that are typically reported (22–63%) [46]. Despite such differences in adherence, the results of our analysis are relevant to real-world practice, as patients less adherent to their daily ICS maintenance tend to rely on additional SABA instead [47]. This is associated with an increased risk of severe exacerbations, and thus related costs including ED visits, hospitalizations and SCS use [48]. A Canadian study found that SABA overuse is associated with a 45% increase in risk of an asthma-related hospital admission, a 25% increase in risk of asthma-related ED visits and a concomitant increase in direct asthma-related costs of 6% [13]. More recent evidence highlights that SABA overuse remains an ongoing issue in the management of patients with asthma in Canada [14, 15]. Approximately 60% of patients with asthma made ≥ 3 SABA canister claims annually in a study that used data from a Nova Scotia healthcare administrative database [14]. This SABA overuse was significantly (p < 0.01) associated with an increased number of asthma-related outpatient visits.

The cost saving associated with as-needed budesonide-formoterol versus low-dose maintenance ICS plus as-needed SABA was driven by lower direct healthcare costs, specifically drug acquisition costs per day, and lower AE costs. This was despite the base-case using a 100% utilization rate of maintenance ICS, i.e., two inhalations per day per the prescribing information. However, to reflect the more realistic utilization rate in SYGMA 2 (there were no maintenance medication reminders [19]), we ran a scenario analysis using the SYGMA 2 ICS utilization rate, and found this did not alter the dominance of as-needed budesonide-formoterol over maintenance ICS plus as-needed SABA. Our decision to derive ICS use from the prescribing information is supported by the Patented Medicine Prices Review Board’s Budget Impact Analysis guidelines, which specify that the dosing frequency used should be according to the product monograph or a public drug plan database, or other applicable drug plan data [41]. CADTH guidelines also state that where costs are directly calculated or imputed, they should reflect the full economic cost borne by the decision-maker [33]. Lower direct AE-related costs in our model could be due to the lower proportion of patients with potential local corticosteroid-related AEs in SYGMA 2 with as-needed budesonide-formoterol [49]. Prior Canadian pharmacoeconomic studies reported that medication costs comprised the largest proportion of annual direct asthma costs (64–68%) [6, 50], which may explain why differences in drug costs underpinned the saving in overall direct costs. AEs were included in our model in part because of recommendations in the CADTH guidelines that health interventions should be assessed in terms of the potential for harms, especially harms that are clinically meaningful and therefore are likely to impact costs [33]. However, as our scenario analysis showed, as-needed budesonide-formoterol was the dominant treatment even without AEs included in the model. Finally, Canada’s publicly funded healthcare system is directed to select treatments for funding by examining their cost and effect trade-off; our study provides evidence of the cost-effectiveness of as-needed budesonide-formoterol as a treatment option for patients with mild asthma.

Given the healthcare system perspective, we did not include indirect costs in the base-case analysis, but these were accounted for in a scenario analysis from the societal perspective. This takes into account the additional burden to the individual and society of time off work, workplace productivity loss [51], functional impairment and caregiver time [52], and out-of-pocket drug costs. When the model was run from the societal perspective, as-needed budesonide-formoterol remained dominant in the cost-effectiveness analysis. These results support previous findings from a Canadian cost-effectiveness study in patients with asthma uncontrolled on maintenance ICS or on maintenance daily ICS-LABA therapy: budesonide-formoterol as maintenance and reliever therapy was shown to be cost-saving on an annual basis from a societal perspective compared with the standard of care [53]. In the current analysis, the total incremental cost saving with as-needed budesonide-formoterol versus low-dose maintenance ICS plus as-needed SABA from the societal perspective was similar to that from the public payer perspective (both $9882). This may be explained, at least in part, by the nature of the societal costs included in this analysis. These were productivity loss, based on days off work per year, which were minimal (0.19 days for all patients in SYGMA 2).

The incremental QALY gain with as-needed budesonide-formoterol was small versus low-dose maintenance ICS plus as-needed SABA treatment. This was likely attributable to using annual severe exacerbations rates as the measure of clinical effectiveness, which were not only similar between the two treatments in SYGMA 2 [19], but also relatively low compared with what would be expected in patients with moderate or severe asthma. Furthermore, had we included mild and moderate exacerbations in the base-case analysis, it is possible that the results would have been even more favorable towards as-needed budesonide-formoterol. Nevertheless, the robustness of our modelling was confirmed by sensitivity analyses.

Limitations of this analysis include those of all Markov models, i.e., that it relies on assumptions, including that transitions between health states are not dependent on the time spent by the patient in any health state or on the sequence of events that preceded it, and that health states are mutually exclusive*.* There was also the potential for ‘double counting’ in some cases of death, as some of the deaths reported in the NRAD study [38] may also have been included in all-cause mortality estimates. While the model was based on a 50-year time horizon, the clinical efficacy data did not extend beyond 1 year of follow-up. Other limitations were the exclusion of mild and moderate exacerbations from the base-case analysis, as well as withdrawal from treatment. However, whilst withdrawal was not modelled in the main analysis, the sensitivity analysis showed it had no real impact. This is because the benefit and costs of both therapies are accrued for as long as they are taken; therefore, low adherence or withdrawal might narrow the difference in costs and QALY between treatment groups but would not change the direction of the results. Notwithstanding these limitations, the various sensitivity and scenarios analyses we conducted confirmed our model was robust.

## Conclusions

From a Canadian public payer perspective, as-needed budesonide-formoterol is a cost-effective treatment over the lifetime of patients with mild asthma for whom the alternative treatment option is low-dose maintenance ICS therapy plus as-needed SABA.

## Supplementary Information

**Additional file 1: Table S1.** Annual risk of death related to severe asthma exacerbation. **Table S2.** Derivation of severe exacerbation costs (Canadian $, 2019). **Table S3.** Annual asthma healthcare resource utilization use and unit costs in the non-exacerbation health state.

## Data Availability

Data underlying the findings described in this manuscript may be obtained in accordance with AstraZeneca’s data sharing policy described at https://astrazenecagrouptrials.pharmacm.com/ST/Submission/Disclosure.

## Abbreviations

AE: Adverse event
CEAC: Cost-effectiveness acceptability curves
CI: Confidence interval
DSA: Deterministic sensitivity analyses
ED: Emergency department
GINA: Global Initiative for Asthma
ICS: Inhaled corticosteroids
LABA: Long-acting β_2_-agonist
NRAD: National Review of Asthma Deaths
PSA: Probabilistic sensitivity analysis
QALY: Quality-adjusted life years
QoL: Quality of life
SABA: Short-acting β_2_-agonist
SCS: Systemic corticosteroid
URTI: Upper respiratory tract infection
WTP: Willingness to pay
