# Supplementary material for: The cost-effectiveness of as-needed budesonide-formoterol versus low-dose inhaled corticosteroid maintenance therapy in patients with mild asthma in Canada
Source: Allergy Asthma Clin Immunol. 2021 Oct 12;17:108. doi: 10.1186/s13223-021-00610-w (PMC8507225; doi:10.1186/s13223-021-00610-w)
Supplement: Supplementary file 1 — Additional file 1: Table S1. Annual risk of death related to severe asthma exacerbation. Table S2. Derivation of severe exacerbation costs (Canadian $, 2019). Table S3. Annual asthma healthcare resource utilization use and unit costs in the non-exacerbation health state. [file 13223_2021_610_MOESM1_ESM.docx]

# Additional file 1:

# The cost-effectiveness of as-needed budesonide-formoterol versus low-dose inhaled corticosteroid maintenance therapy in patients with mild asthma in Canada

**Mohsen Sadatsafavi,^1^ J Mark FitzGerald,^2^ Paul M O’Byrne,^3^ Mena Soliman,^4^ Niroshan Sriskandarajah,^5^ Colin Vicente,^6^ Sarowar Muhammad Golam^7^**

^1^Respiratory Evaluation Sciences Program, Collaboration for Outcomes Research and Evaluation, Faculty of Pharmaceutical Sciences, University of British Columbia, 2405 Wesbrook Mall, Vancouver, BC, V6T1Z3; Canada; msafavi@mail.ubc.ca (corresponding author)

^2^Centre for Lung Health, Vancouver Coastal Health Research Institute, University of British Columbia, 2775 Laurel Street, Vancouver, BC V5Z1M9, Canada; Mark.Fitzgerald@vch.ca

^3^Firestone Institute of Respiratory Health, St Joseph’s Healthcare and Department of Medicine, Michael G. DeGroote School of Medicine, McMaster University, 1280 Main Street West, Health Science Center, 3W10 Hamilton, Ontario L8S 4K2, Canada; obyrnep@mcmaster.ca

^4^Medical Affairs, AstraZeneca, 1004 Middlegate Road, Mississauga, Ontario L4Y 1M4, Canada; mena.soliman@astrazeneca.com

^5^Market Access, AstraZeneca, 1004 Middlegate Road, Mississauga, Ontario L4Y 1M4, Canada; niroshan.sriskandarajah@astrazeneca.com

^6^PIVINA Consulting Inc., 2600 Skymark Avenue, Suite 11-202, Mississauga, ON, L4W 5B2, Canada; cvicente@pivina.com

^7^Global Market Access and Pricing, BioPharmaceuticals R&D, AstraZeneca, 431 83 Mölndal, Gothenburg, Sweden; sarowar.golam@astrazeneca.com

**Table S1.** Annual risk of death related to severe asthma exacerbation.

| **Type of severe exacerbation** | **Patient age, years** | **Annual risk of death** | **Source** |
| --- | --- | --- | --- |
| SCS | 12–16 | 0.0004 | Watson *et al.* 2007^1^  NRAD 2017^2^ |
|  | 17–44 | 0.0005 |  |
|  | 45–54 | 0.0032 |  |
|  | 55–64 | 0.0032 |  |
|  | 65–100 | 0.0032 |  |
| ED visit + SCS | 12–16 | 0.0027 | Watson *et al.* 2007^1^  NRAD 2017^2^ |
|  | 17–44 | 0.0032 |  |
|  | 45-54 | 0.0206 |  |
|  | 55–64 | 0.0206 |  |
|  | 65–100 | 0.0206 |  |
| Inpatient hospitalization | 12–16 | 0.0014 | Watson *et al.* 2007^1^  Roberts *et al.* 2013^3^ |
|  | 17–44 | 0.0020 |  |
|  | 45–54 | 0.0076 |  |
|  | 55–64 | 0.0214 |  |
|  | 65–100 | 0.0454 |  |

ED: emergency department; NRAD: National Review of Asthma Deaths; SCS: systemic corticosteroid.

**Table S2**. Derivation of severe exacerbation costs (Canadian $, 2019).

| **Severe exacerbation types** | **HCRU related to exacerbation** | **Unit cost ($)** | **Source** | **Mean duration (days)** | **Total cost per event ($)** |
| --- | --- | --- | --- | --- | --- |
|  |  |  |  | **SYGMA 2** |  |
| **SCS** | SCS (day) | 0.00 | Assumed covered by ED or hospital cost | 6.48 | **155.14** |
|  | GP visit - prescribing | 77.20 | Ontario Schedule of Benefits^4^ | 1 |  |
|  | GP visit- follow-up | 77.20 | Ontario Schedule of Benefits^4^ | 1 |  |
| **ED visit +**  **SCS** | ED visit (visit) | 412.87 | <https://odprn.ca/wp-content/uploads/2015/04/ICS-LABA-asthma-Final-Pharmacoeco-04-03-15_AS-srk.pdf> | 1 | **490.81** |
|  | SCS (day) | 0.00 | Assumed covered by ED or hospital cost | 5.53 |  |
|  | GP visit Follow-up | 77.20 | Ontario Schedule of Benefits^4^ | 1 |  |
| **Inpatient hospitalization** | HRG stay (LOS=6 days) (stay) | 9,322.00 | OCCI Code J441^5^ | 1 | **9,399.94** |
|  | Additional day (day) | N/A | N/A | N/A |  |
|  | GP visit Follow-up | 77.20 | Ontario Schedule of Benefits^4^ | 1 |  |

ED: emergency department; GP: general practitioner; HRG: healthcare resource group; HCRU: healthcare resource utilization; LOS: length of stay; N/A: not applicable; OCCI: Ontario Case Costing Initiative; SCS, systemic corticosteroid.

**Table S3.** Annual asthma HCRU use and unit costs in the non-exacerbation health state.

| **Type of medical resource** | **Unit cost ($)*** | **HCRU per patient-treatment year†** | **Source for unit costs** |
| --- | --- | --- | --- |
| **Specialist visit** | 157.00 | 0.16 | Ontario Schedule of Benefits^4^ |
| **Primary healthcare physician visit** | 77.20 | 0.1 | Ontario Schedule of Benefits^4^ |
| **Telephone calls to physician** | 10.00 | 0.09 | Assumed as per nurse calls |
| **Ambulance transport** | 168.40 | <0.01 | Ontario Schedule of Benefits^4^ |
| **Home visits by physician** | 77.20 | <0.01 | Assumed as per primary visit |
| **ED visit (not captured as exacerbation)** | 97.60 | 0.01 | Ontario Schedule of Benefits^4^ |
| **Other healthcare visit** | 77.20 | 0.03 | Assumed as per primary visit |
| **Home visits/other healthcare** | 40.00 | <0.01 | 1 hour per visit Registered Nurses’ Association of Ontario^6^ |
| **Telephone calls to nurse** | 10.00 | 0.02 | 15 minute per call Registered Nurses’ Association of Ontario^6^ |

ED: emergency department; HCRU: healthcare resource utilization.

*The most recent Ontario Schedule of Benefits was published in 2017; costs were unchanged at the time this cost-effectiveness analysis was conducted, in May 2019.

^†^In the base-case, non-exacerbation health state costs were calculated by taking HCRU data from SYGMA 2 and multiplying by standard specific unit costs.

**References**

1. Watson L, Turk F, James P, Holgate ST. Factors associated with mortality after an asthma admission: a national United Kingdom database analysis. Respir Med. 2007;101:1659-64.
2. Royal College of Physicians. Why asthma still kills. The National Review of Asthma Deaths (NRAD). <https://www.rcplondon.ac.uk/projects/outputs/why-asthma-still-kills> (2014). Accessed 30 Mar 2020.
3. Roberts NJ, Lewsey JD, Gillies M, Briggs AH, Belozeroff V, Globe DR, et al. Time trends in 30 day case-fatality following hospitalisation for asthma in adults in Scotland: a retrospective cohort study from 1981 to 2009. Respir Med. 2013;107:1172-7.
4. Ontario Health Insurance Plan (OHIP). OHIP Schedule of Benefits and Fees. <http://www.health.gov.on.ca/en/pro/programs/ohip/sob/> (2017). Accessed 6 Mar 2020.
5. Ontario Ministry of Health and Long-Term Care (MOHLTC) - Health Data Branch. Ontario Case Costing Initiative (OCCI). <https://hsim.health.gov.on.ca/hdbportal/> (2019). Accessed 6 Feb 2020.
6. Registered Nurses Association of Ontario. Dollars and Sense: What are nurses paid? <http://careersinnursing.ca/new-grads-and-job-seekers/find-nursing-job/dollars-and-sense-what-are-nurses-paid> (2020). Accessed May 2019.
